# Frequency of “Nursing Strike” among 6-Month-Old Infants, at East Tehran Health Center and Contributing Factors 

**Published:** 2015-09

**Authors:** Fatemeh Nayyeri, Farima Raji, Edith Haghnazarian, Mamak Shariat, Hosein Dalili

**Affiliations:** 1 Breast feeding Research Center, Tehran University of Medical Sciences, Tehran, Iran; 2 Maternal, Fetal Neonatal Research Center, Tehran University of Medical Sciences, Tehran, Iran

**Keywords:** Nursing strike, Breastfeeding, Infant, Tehran

## Abstract

**Objective:** An abrupt refusal by the infant to breastfeed is often called “nursing strike”. In fact a common reason for cessation of nursing is infant’s refusal to breast feed. This problem can often be overcome. This paper has aimed to identify the causes of “breast feeding refusal” or “nursing strike” in 6 month old infants visiting the East Tehran health center for their scheduled vaccination of 6 months old.

**Materials and methods:** Totally 175 six month old infants were enrolled in this study. A questionnaire was filled by mother for each child and later the infants with “nursing strike” were compared with all others.

**Results:** In this study prevalence of breast feeding refusal in infants was 24%.There was significant relation between the “breastfeeding refusal” and maternal academic education or working status. In this study mothers reported various reasons associated with “refusal breast feeding. According to the mothers playful infant and nasal obstructions were the probable causes for refusal.

**Conclusion:** There is a diverse variety of factors influencing nursing strike. Most of these factors can be prevented by identifying the background reasons and proper training.

## Introduction

An abrupt refusal by the infant to breastfeed is often called “nursing strike” (1, 2). The baby’s refusal to breastfeed can be a stressful and concerning matter for a mother. The mother might take this personally and believe her child is refusing her and not the breast milk. She might also think that there is something missing from her milk and it’s insufficient for her child (1, 2). In fact a common reason for cessation of nursing is infant’s refusal to breastfeed. But this problem can often be overcome (3). Having the knowledge and focusing on this problem can prolong breastfeeding.

The mother should be reassured that there is definitely a reason behind the baby’s attitude and by acknowledging it she can encourage her newborn to re-breastfeed (4). In order to help the mother and her child in surmounting this problem and to restart mother-infant connection we ought to dig deeper into the possible reasons behind this.

Breastfeeding strike can happen in any age group and any period of life and each have different causes (1-3).

The reasons can be classified into 3 categories: infant related factors, mother related factors and milk production related factors.

1. Infant related factors: can be respiratory, infectious, teething, gastro-esophageal reflux, separation, nasal obstruction, cerebral injuries and pain (3, 4).

2. Mother-related factors: can also be responsible for the refusal such as mastitis, change in the nursing pattern and drugs

3. Slow or insufficient milk production has also been mentioned as another reason for refusal to breastfeed (3, 4).

Little literature exists on “nursing strike”. Knowing the proven benefits of breastfeeding in preventing malnutrition, especially in developing countries, and by considering the fact that this is a solvable problem, we therefore conducted a study to evaluate the most probable causes for “nursing strike” and the overall frequency of it. This study was done on 6 month old infants visiting East Tehran health center.

## Materials and methods

In the current study, we tried to identify the causes of “breast feeding refusal” or aforementioned “nursing strike” in 6 month old infants visiting the East Tehran health center for their scheduled vaccination of 6 months old.

A questionnaire was prepared which included items such as: the exact age at which the strike started, possible causes (believed by the mothers), mothers’ demographic data, gestational history, prenatal education, known underlying diseases in the mother, infants’ demographic data, any illnesses in the child.

Exclusion criteria consisted of: infants with known congenital malformation, a history of prematurity and having an underlying disease. 

This study was approved by research committee of Tehran University of Medical Sciences under the reference number of: 90-03-105-14890. After explaining the goals of the study to the mothers, 175 six month old infants visiting for vaccination were enrolled in this study. A questionnaire was filled for each child, and later the infants with “nursing strike” were compared with all others. Health professionals explained the details to the mothers and filled the questionnaires according to the mothers’ responses.

The data were recorded and analyzed by SPSS software V.16 for Windows. For the quantitative data the student T-test was used and for the qualitative data the Chi-square was performed. Significance level was set on 95% for all variables.

## Results

Tables 1 and 2 demonstrate the mother and the infant’s demographic data. Total frequency of “nursing strike” in 6-month old infants was 41 out of 175 (24%). The mean age of “breastfeeding refusal” was 5.28 ± 1 month (with a range of 2- 6.5 months). Table 3 demonstrates the relation between the measured factors and “breastfeeding refusal”. There was no significance relation between the “breastfeeding refusal” and gestational age, type of delivery, having previous nursing education, and maternal underlying conditions. Although the relationship with maternal academic education or working mothers was significant, meaning there was more “breastfeeding refusal” in the working mothers or those with higher education.

**Table 1:** Mothers’ demographic data

Age (year) (mean ± SD)	30.20 ±5.35
Education [n (%)]	
Graduated	98 (70%)
Undergraduate	43 (30%)
Occupation [n (%)]	
Working	10 (9%)
Stay at home	98 (91%)
Parity(mean ± SD)	1.52 ± 0.677
Underlying disease [n (%)]	
Yes	23 (15%)
No	120 (85%)

**Table 2:** Neonates’ demographic data

Sex	
Male	85 (49%)
Female	89 (51%)
Birth weight (grams) (mean± SD)	3444± 231
Head circumference (centimeter) (mean ±SD)	34.71 ± 2.20
Height (centimeter) (mean ± SD)	49.80± 2.48
Gestational age (weeks) (mean± SD)	38.98± 1.35

According to the mothers the probable causes for refusal were: playful infant (50%), nasal obstruction (31%), vaccination area pain (19%), flat nipple (14.3%), breast engorgement (11.9%), labor injuries (9.5%) and teething (4.8%).

**Table 3:** Relationship between nursing refusal and relevant factors

**Mother’s charectristics**	**Neonates with refusal to breast feed**	**Neonates without refusal**	**p-value**
Education [n (%)]			0.01
Undergraduate	8 (17%)	35 (83%)	
Graduated	35 (36%)	63 (64%)	
Occupation [n (%)]			0.04
Stay at home	39 (34%)	95 (66%)	
working	4 (40%)	6 (60%)	
Type of delivery [n (%)]			0.58
C/S	28 (27%)	75 (73%)	
NVD	13 (32%)	28 (68%)	
Underlying disease			0.20
Yes	5 (22%)	18 (78%)	
No	38 (32%)	82 (68%)	
Breast feeding education			0.45
Yes	34 (30%)	78 (70%)	
No	7 (22%)	23 (78%)	
Gestational age (mean± SD)	39 ± 1.31	38 ± 1	0.06

## Discussion

Factors attributed to or associated with nursing strike can be classified into 3 categories: Medical (maternal and infantile conditions), Psychological (playful child), and social (mother-infant separation). The current study indicates that the psychological factors are observed in many cases and most of other factors account for unsuccessful exclusive breastfeeding as well.

In this study mothers reported various reasons associated with “refusal breast feeding” (in order of frequency): a playful child, nasal obstruction, vaccination area pain, flat nipple, breast engorgement, labor traumas and teething. The reason the study emphasized on the age of 6 months was the importance exclusive breast feeding by the age of 6 months.

WHO classifies the causes of nursing strike into 4 categories: 1.pain or illness 2.breast feeding technique 3.change in newborn’s care and 4.True refusal, each of these categories involves several items. 

Causes mentioned in this study also fit in these categories. But because Only 6 month olds were studied in this study and WHO has studied the entire period of infancy, some of the causes were not achieved. “Refusal to nurse” often has a sudden onset, but with a change in conception and understanding of the matter it can easily be overcome (5).

In a study by Li R. breast feeding refusal in infants below 1 year old was 47.3% compared to ours which was 24%, this difference can be because of the limited age group in our study (6).

Another study indicated that at the age of 4 month the baby gets easily distracted and does not focus on the mother’s face as much. The infant comes off the breast to look around (7); our results similarly showed that the most prevalent reason for “refusal to nurse” is the baby’s distractibility and playfulness, whereas this reaction by the infant can be a sign of unwillingness or refusal in some mothers (especially primigravidas).

A different study added other associated factors such as pain, breast problems, oral disorders, prematurity, Gastroesophageal reflux disease, infantile colic, allergy, cleft palate, and sublingual nodes (8).The current study had excluded prematurity and congenital malformations. One study has mentioned Herpes as one of the causes for refusal, but in the current study none of the mothers were actively or previously infected by the virus (9).

In the current study, “Nursing strike” was significantly higher in working mothers which is probably due to change in baby’s care. It should also be mentioned that in WHO’s guidelines one of the causes of refusal in 3-12 month old infants is mother-infant separation and having new Nurse (3).

There was a significant relationship between “refusal of breast feed” and mother's education, and refusal was surprisingly higher in infants of the mothers with higher education, and it could be because these mothers mostly have a job outside the house. Most of these causes can easily be prevented by proper education to the mothers. For example the pregnant mothers should be aware of common problems during nursing such as breast problems, infantile illnesses, psychological and behavioral matters. It has been proven that postpartum home visits can remarkably reduce nursing refusal (10).

WHO has developed new methods to successfully initiate and continue breast feeding. One of them is to avoid using artificial nipples and pacifiers for nursing infants. Using a pacifier instead of the breast to soothe the baby can reduce breastfeeding, subsequently milk production and eventually shorten breastfeeding period. This theory can be a possible reason behind the fact that there is more refusal in working and highly educated mothers, as the babysitters mostly use pacifier to calm the baby.

There is a diverse variety of factors influencing breastfeeding refusal or nursing strike. Most of these factors can be prevented by identifying the background reasons and proper training. Examples are breastfeeding in a quiet and calm environment with as few stressors as possible, and holding (to increase skin to skin contact) (1). More studies are needed in this area.
